# Laser-Induced Synthesis of Electrocatalytically Active Ag, Pt, and AgPt/Polyaniline Nanocomposites for Hydrogen Evolution Reactions

**DOI:** 10.3390/nano13010088

**Published:** 2022-12-24

**Authors:** Anna A. Vasileva, Daria V. Mamonova, Yuri V. Petrov, Ilya E. Kolesnikov, Gerd Leuchs, Alina A. Manshina

**Affiliations:** 1 Saint Petersburg State University, 26 Universitetskii Prospect, 198504 Saint-Petersburg, Russia; 2 Max Planck Institute for the Science of Light, Staudtstr. 2, 91058 Erlangen, Germany; 3 Friedrich-Alexander-Universität Erlangen-Nürnberg, Staudtstr. 7/B2, 91058 Erlangen, Germany

**Keywords:** laser-induced deposition, polyaniline, noble metal nanoparticles, AgPt nanoparticles, nanocomposite, hydrogen evolution reaction

## Abstract

We present an efficient and easily implemented approach for creating stable electrocatalytically active nanocomposites based on polyaniline (PANI) with metal NPs. The approach combines in situ synthesis of polyaniline followed by laser-induced deposition (LID) of Ag, Pt, and AgPt NPs. The observed peculiarity of LID of PANI is the role of the substrate during the formation of multi-metallic nanoparticles (MNP). This allows us to solve the problem of losing catalytically active particles from the electrode’s surface in electrochemical use. The synthesized PANI/Ag, PANI/Pt, and PANI/AgPt composites were studied with different techniques, such as SEM, EDX, Raman spectroscopy, and XPS. These suggested a mechanism for the formation of MNP on PANI. The MNP–PANI interaction was demonstrated, and the functionality of the nanocomposites was studied through the electrocatalysis of the hydrogen evolution reaction. The PANI/AgPt nanocomposites demonstrated both the best activity and the most stable metal component in this process. The suggested approach can be considered as universal, since it can be extended to the creation of electrocatalytically active nanocomposites with various mono- and multi-metallic NPs.

## 1. Introduction

The hydrogen evolution reaction (HER) plays a critical role in electrochemistry and has attracted attention for both application and fundamental research. This reaction is one of the most important in developing new technologies for creating environmentally friendly energy sources [1,2]. Moreover, the hydrogen evolution reaction is useful as a test reaction for the development of new types of catalysts. As a catalyst for HER, mono- and multi-metallic nanoparticles (MNPs) have been widely studied [3,4]. In spite of the research devoted to the creation of catalysts on the basis of less expensive metals such as Ni, Cu, Fe, etc. [5,6,7,8], noble metal (mainly Pt) catalysts are still preferred. One way of improving catalysts’ efficiency is related to the use of metal nanostructures with different shapes [9]. Another approach is based on the use of bimetallic NPs [10,11,12,13]. This approach relies on the redistribution of the local electron density on the surface layer of NPs, which leads to less destruction of the catalysts by changing the reaction kinetics. Therefore, diversifying the strategies used for the synthesis of various multi-metallic NPs with electrocatalytical potency remains an urgent scientific problem. Up to now, different combinations of metals have displayed HER activity, such as PtCo [13], PtBi [14], PtIr/IrOx [15], PtRu [16], and RhRt [17]; however PtAg systems are of high interest because of the good miscibility of these metals and their selective corrosion in an acidic medium, caused by a large difference in their standard redox potentials [18]. Altogether, this results in less corrosion of PtAg systems during HER reaction.

The redox properties of polyaniline (PANI) make it an attractive material for creating electrochemical devices based on it. The addition of MNPs to the system significantly expands the range of possible applications such as sensors [19,20,21], supercapacitors [22,23,24], and fuel cells [25,26]. Combining PANI electrodes with metal nanoparticles has proven their efficacy in the development of sensors for hydrogen peroxide [27], glucose [28,29], nitrate ions [30], etc. In such systems, PANI often plays the role of a conducting matrix, while the electrocatalytic reaction occurs directly on the MNPs. Taking this into account, the rational choice of the metal NPs provides the final functionality of the electrode.

Polyaniline can be considered as a promising competitor to carbon nanotubes, graphene oxide, reduced graphene oxide, Nafion, etc., as a prospective matrix for electrode systems due to its conductivity, high chemical stability, high material porosity, and the possibility of surface functionalization. The combination of these features is promising for creating stable PANI/MNP nanocomposite electrodes because of the possibility of PANI interacting with MNPs and its effect on the reaction kinetics. In spite of PANI’s prospects as nanocomposite matrix, there are still several challenges in connection with the incorporation of catalytically active nanoparticles onto the surface of PANI.

Nowadays, wet chemistry methods are widely used for the creation of various MNPs. However, these approaches require a further step of immobilization of the synthesized NPs on the electrode’s surface. For immobilization, drop-casting or spin-coating methods are typically used. These methods are attractive because of their experimental simplicity, but the obtained samples demonstrate weak adhesion to the substrate. As an alternative, one could consider electrochemical methods of synthesizing multi-metallic NPs, but this is a multi-step process because the deposition of different metals requires the application of different voltages. In most cases, the main problem limiting the practical use of the newly developed nanocomposite electrodes is the loss of catalytically active particles from the electrode’s surface. Therefore, the development of an efficient strategy for the creation of stable PANI/MNP nanocomposite electrodes based on bimetal NPs with electrochemical potency is a current scientific task. In the current work, we suggest an advanced approach for obtaining electrocatalytically active nanocomposite electrodes based on PANI synthesized by in situ oxidative polymerization followed by a one-step laser-induced synthesis of MNPs with simultaneous immobilization on PANI.

Laser-induced deposition (LID) is an attractive method for synthesizing NPs. The method is based on the photochemical decomposition of the metal-containing precursor under laser irradiation, followed by reduction of the metal and the formation of NPs [31]. As possible precursors, an extensive list of laboratory-synthesized and commercially available compounds were successfully demonstrated, such as organometallic complexes, inorganic, and organic salts [32,33,34,35,36,37,38,39]. It was found that LID allows the user to control the coating density, morphology, and composition of the MNPs deposited on the substrate’s surface. To date, various mono-, bi-, and trimetallic nanoparticles (Ag, Au, Pt, Cu, Ru, AgPt, and AgAuPt) have been deposited on 2D and 3D amorphous, crystalline, and polycrystalline substrates [35,40,41,42].

The main attractiveness of LID for electrochemical applications is the good adhesion of NPs to the substrate, as the nucleation and growth of NPs is a heterogeneous laser-induced process taking place directly on the surface of the substrate. Another important feature of the LID method is the use of low-intensity laser radiation, which avoids the decomposition of the NPs and substrate. because of the abovementioned factors, the aim of the current research was laser-induced deposition of MNPs on the surface of a polymer substrate (PANI) and the subsequent characterization of the obtained PANI/MNP nanocomposites as electrodes for HER. NPs of Ag, Pt, and their combination were chosen. The LID synthesis of NPs on polyaniline surfaces and the electrochemical characterization of the PANI/MNP nanocomposite are presented here for the first time. We demonstrated that the LID of MNPs onto a polymer’s surface not only is possible but also allows the interaction of PANI and MNPs, which is of great importance for the creation of stable functional nanocomposites for electrochemical applications.

The high interest in studying multicomponent systems is determined by the wide range of various processes within the system, resulting in new physicochemical and functional properties. Moreover, such systems are characterized by the synergistic effect of the components’ interactions. The novelty of the present work is provided by its consideration of a multicomponent system, i.e., conductive polymer PANI and catalytically active metals (Ag and Pt). This multicomponent system was examined in different combinations, namely Ag/PANI, Pt/PANI, and AgPt/PANI. Such an approach allowed us to observe and analyze complex processes in multicomponent systems with electrocatalytical potency, which have not been studied before. Consistent consideration of these systems from a unified standpoint made it possible to conduct a thorough analysis of the features of the interfacial interactions in terms of their chemical structure and functionality (electrocatalysis). The chosen approach allowed us to discover the processes that are important for the functioning of the nanocomposites as electrocatalytically active systems. The observed processes were (i) the effect of PANI in the formation of Pt NPs in the course of LID; (ii) the formation of bimetallic PtAg NPs, which is a complex process initiated by LID of the Ag phase and followed by galvanic Pt replacement; (iii) LID of MNPs allowed a PANI–MNP interaction. All the observed characteristics of the studied multicomponent system are important for the creation of stable electrocatalytically active nanocomposites.

## 2. Experimental procedures

### 2.1. Materials and Methods

Menzel cover slips with a size of 20 mm x 20 mm were used as substrates. The electrical contacts were formed from gold (99.99%). The chemicals were purchased from Alfa Aesar, (Ward Hill, USA) and Reachem (Saint-Petersburg, Russia). For the synthesis of the polyaniline, doubly distilled water, chloric acid, ammonium persulfate, and aniline were used. The aniline was purified preliminarily by a standard procedure. The ammonium persulfate was kept in darkness in the presence of desiccant and used as received. The aniline was purified before synthesis according to a standard procedure [43]. For LID, the following reagents were used as metal precursors: silver benzoate (silver salt C_6_H_5_COOAg, containing Ag 47.1 wt%) and tetra-ammine platinum(II) hydroxide (Pt(NH_3_)_4_(OH)_2_, containing Pt 52.8–63.7 wt%). The solvent was doubly distilled water. A 0.1 M HClO_4_ solution was used for the electrochemical measurements. Polyimide Kapton tape was used for preparation of the masks.

### 2.2. PANI Synthesis

The cover glasses were purified with isopropanol by ultrasound treatment for 5 min and dried in flowing air. Then glasses were covered with 10 nm of Ti and 25 nm of Au by a resistive evaporation method in a vacuum. Next, the substrates were used for in situ PANI synthesis, for which we diluted 5.71 g of the ammonium persulfate solution in 50 mL of water and 1.86 mL of aniline in 50 mL of 0.2 M HCl. The solutions were shaken, and the temperature was stabilized at 25 °C for 1 h. Next, 300 µL of each solution was placed in an Eppendorf tube, rapidly mixed, and immediately dropped on the substrate. In situ PANI synthesis took 30 min. After synthesis, the substrate was cleaned in flowing 0.2 M HCl and isopropanol, then additionally with 0.2 M HCl. The samples were dried overnight under ambient conditions.

### 2.3. LID

The scheme of the LID experiment has been presented elsewhere [44]. In the current work, we used the following compounds as precursors for LID: silver benzoate and tetra-ammine platinum (II) hydroxide. LID was performed with aqueous solutions of silver benzoate (0.6mg/mL) and tetra-ammine platinum (II) hydroxide (0.5 mg/mL). The concentrations of the precursors were chosen accordingly to their solubility. For the deposition of bimetallic AgPt NPs, mixed aqueous solutions of silver benzoate and tetra-ammine platinum (II) hydroxide were prepared. For the preparation of the solutions, a certain amount of the precursor was diluted in water and ultrasonicated for 5 min. Next, 200 µL of the solution was dropped to the PANI substrate, and the sample was placed under a laser beam for 40 min. As the laser source, a continuous wave MBD-266 laser (from Coherent, Santa-Clara, USA) with a wavelength of 266 nm was chosen. The laser beam was defocused to a diameter of ~1 cm, and the laser’s power density was 15 mW/cm^2^. Such a low intensity allowed us to avoid decomposition of the PANI. As a result of the LID process described here, monometallic Ag and Pt NPs, and bimetallic AgPt NPs were obtained on the surface of the polyaniline.

### 2.4. Sample Characterization

The samples’ morphology was investigated using a scanning electron microscope (SEM) Zeiss Merlin (from Karl Zeiss, Oberkochen, Germany) equipped with a field emission cathode, a GEMINI-II electron-optics column, and an INCAx-act energy dispersive X-ray spectrometer (EDX), all in an oil-free vacuum system (Oxford Instruments, Abingdon, UK).

The samples’ structure was investigated by Raman spectroscopy using a confocal spectrometer Senterra (Bruker, Billerica, MA, USA). The Raman spectra were excited by a 785-nm solid-state laser (1 mW power) using a 20× objective with a 200 s acquisition time, and the spectra were collected four times. For each sample, we recorded the spectra in three different areas of the sample and presented the average spectrum.

In addition, XPS measurements were performed using a photoelectron spectrometer (Escalab 250Xi, Waltham, MA, USA) with AlKα radiation (photon energy = 1486.6 eV). Spectra were recorded in the constant pass energy mode at 100 eV for the survey spectrum and at 50 eV for the element core level spectra; the XPS spot size was 650 μm.

### 2.5. Electrochemistry

Electrochemical measurements were performed in a three-electrode cell with the sample operating as a working electrode, an Ag/AgCl (KCl_3M_) electrode featuring a redox potential of +0.21 V versus a standard hydrogen electrode (SHE) as a reference electrode, and a Pt mesh as a counter-electrode. Measurements were carried out with potentiostat/galvanostat Gamry Reference 600 (Gamry Instruments, Warminster, USA). Measurements were carried out in 0.1 M perchloric acid. The cyclic voltammetry (CV) measurements were recorded at V_scan_ = 50 mV/s; the third cycles are presented unless otherwise specified. The impedance spectroscopy (EIS) measurements were performed across a range from 100 kHz to 0.01 Hz in potentiostatic mode with the application of the peak potential for the studied process or at −0.5 V otherwise. The amplitude of the applied sinusoidal voltage was ±10 mV. The stability tests were performed by continuous cycling (100 cycles) and chronoamperometry for 1 h at the chosen voltage (as specified below).

## 3. Results and Discussion

### 3.1. Characterization of the Samples

**Figure 1:**
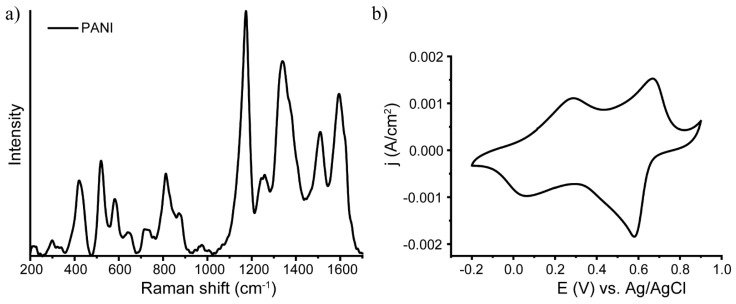
Characterization of the PANI synthesized by in situ polymerization: (**a**) Raman spectra; (**b**) cyclic voltammetry.

As a first step, polyaniline was synthesized by in situ oxidative polymerization, as developed earlier by our group [45]. As a result of the synthesis, a green film on the substrate was obtained. The synthesized polymer was characterized by Raman spectroscopy. The results are presented in Figure 1a.

The Raman spectrum allowed us to identify the synthesized polymer as polyaniline in the form of emeraldine salt [46,47,48]. The detailed interpretation of the observed peaks was in accordance with the published data and is presented in Table 1.

The obtained PANI films were also characterized by cyclic voltammetry; the results are presented in Figure 1b. As shown in the figure, there are two pairs of waves in the CV curves, which correspond to two types of redox processes. One pair of waves in the region of higher potential (maximum at +0.6 V in the cathodic curve, minimum at +0.7 V in the anodic curve) corresponds to the emeraldine–pernigraniline transition, and the pair in the region of lower potential (+0.05 V in the cathodic curve, +0.25 V in the anodic curve) corresponds to the leucoemeraldine–emeraldine transition [55]. Both Raman spectroscopy and cyclic voltammetry confirmed the formation of polyaniline in the form of emeraldine salt.

**Figure 2:**
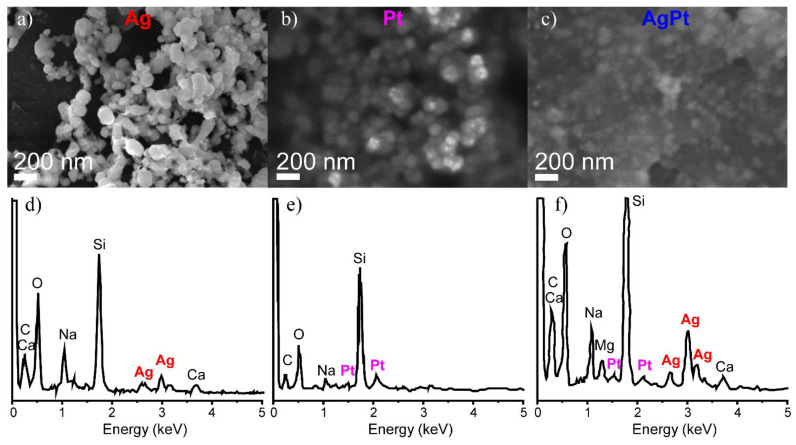
SEM images of LID on PANI with (**a**) C_7_H_5_AgO_2_, (**b**) Pt(NH_3_)_4_(OH)_2_, and (**c**) C_7_H_5_AgO_2_+Pt(NH_3_)_4_(OH)_2_ solutions. EDX analysis of LID on PANI with (**d**) C_7_H_5_AgO_2_, (**e**) Pt(NH_3_)_4_(OH)_2_, and (**f**) C_7_H_5_AgO_2_+Pt(NH_3_)_4_(OH)_2_ solutions.

Next, laser-induced deposition of metal NPs on the PANI was performed. In the first stage, adaptation of the LID method to polyaniline surfaces was carried out. It was necessary to find appropriate parameters for laser irradiation to avoid destroying the polymer. The destruction of PANI may be caused by local heating of the substrate as a result of PANI absorbing the laser’s radiation and its reflection from the gold layer below. The absence of destruction was checked by the Raman spectra recorded before and after irradiation. If there were no changes in the PANI spectrum after laser irradiation, the polymer was considered stable under the level of laser radiation used. The polymer was found to be stable under laser irradiation with a power density of up to 15 mW/cm^2^. The intensity of 15 mW/cm^2^ was used for further LID experiments on PANI. The SEM and EDX data for the NPs deposited on PANI are displayed in Figure 2.

The data presented here confirmed the formation of NPs on PANI (Figure 2a–c). The brighter areas in the SEM images are associated with metal NPs and the darker areas are associated with the polymer. In case of LID with the C_7_H_5_AgO_2_ solution, NPs with sizes of up to 100 nm could be observed (Figure 2a). EDX analysis confirmed the formation of silver NPs (Figure 2d). In the case of deposition with the Pt(NH_3_)_4_(OH)_2_ solution, one can observe smaller NPs (30–50 nm) with a high degree of agglomeration. The diffusivity of the SEM image may be associated with the high agglomeration of metal NPs on the PANI film (Figure 2b). The EDX analysis confirmed that in this case, Pt NPs were deposited (Figure 2e). In contrast, for deposition with a mixed solution of silver and platinum precursors, the obtained NPs (~40 nm) were uniformly distributed on the PANI matrix; EDX confirmed the presence of both metals (Figure 2c,f). Our previous results on the deposition of multi-metallic nanoparticles by LID revealed the formation of structures representing monometallic crystallites and areas of alloys of the respective metals [42]; this allowed us to assume the formation of similar AgPt structures.

**Figure 3:**
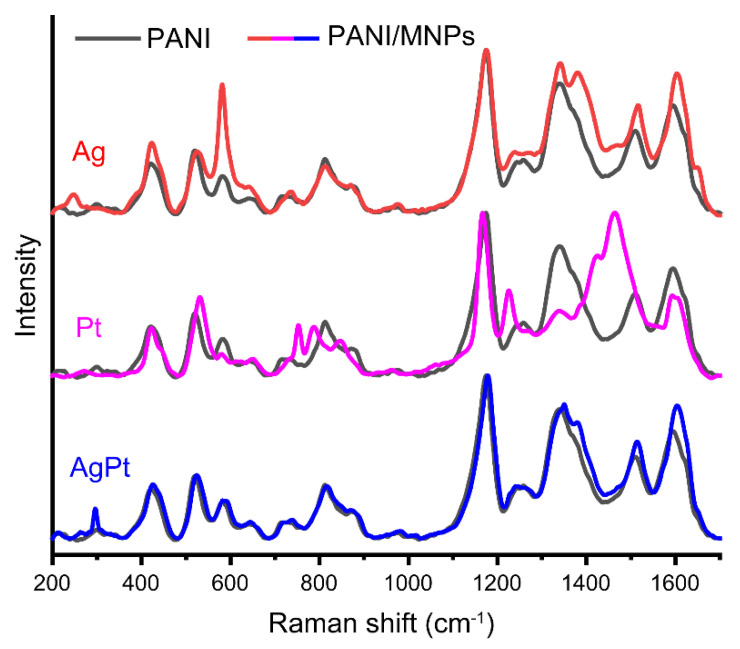
Raman spectra for pure PANI (black lines) and PANI/MNP (Ag, Pt, AgPt) composites (red, magenta and blue lines, respectively).

To study the role of PANI in the LID process, the structural changes in PANI as a result of the deposition of Ag, Pt, and AgPt NPs were investigated by Raman spectroscopy (Figure 3).

Changes in the Raman spectra of PANI as a part of the nanocomposites in comparison with pure PANI were observed. It is interesting to note that the PANI/AgPt sample had a minimal difference from the structure of pure PANI. The main difference can be found in the spectral region at about 1300–1500 cm^−1^.

Moreover, for the samples with monometallic NPs, there were changes in the spectral ranges at about 600 and 800 cm^−1^. In case of PANI/Ag, the intensity of the peak at 572 cm^−1^ increased significantly. This peak is related to the vibration of phenoxazine-type units (Table 1). The increase in the intensity of the phenoxazine-related peak may be related to the redistribution of the electron density caused by the attraction of silver by oxygen. This assumption was confirmed by the appearance of a peak at 248 cm^−1^, which is assigned to the vibration of Ag-O formed as a result of the adsorption of silver by oxygen-containing fragments [56].

One can also observe the growth in the band’s intensity at 1375 cm^−1^ for PANI/Ag. This testifies to an increase in the contribution of bipolaron to the structure of PANI after the deposition of Ag. The peak related to N-H deformation in the semiquinonoid structures shifted from 1509 to 1511 cm^−1^, and the peak related to the C–C stretching vibration in the semiquinonoid structures shifted from 1594 to 1600 cm^−1^. These changes in the spectra testify to a slight shift in the electron density between NPs and the PANI substrate, and may be also assigned to the adsorption of NPs on PANI.

In case of PANI/Pt, the spectrum of the composite differed significantly from the spectrum of pure PANI. There was a shift from 520 to 533 cm^−1^ (out-of-plane ring deformation), a shift from 720 to 752 cm^−1^ (deformation of amine fragments), a shift from 808 to 790 cm^−1^ (out-of-plane C-H deformation of the quinonoid ring), a shift from 865 cm^−1^ to 849 cm^−1^ (substituted benzene ring deformations), a shift from 1254 to 1227 cm^−1^ (C–N stretching in quinonoid structures), and a decrease in the peak intensity of polaron at 1336 cm^−1^. The band with maxima at 1424 and 1456 cm^−1^ may be interpreted as the result of the shift and the increase in the intensity of the bipolaronic band (1375 cm^−1^) and the band related to N-H deformation in the semiquinonoid structures (1509 cm^−1^). Such significant changes in the spectra testify to the partial modification of PANI as a result of the deposition of Pt NPs. Mainly, the peaks associated with nitrogen atom bonds shifted, which testifies to the change in the surrounding nitrogen and weakening of the bonds in PANI. This allowed us to assume that the nitrogen in PANI acted as a reducing agent for the platinum ions in the process of NPs forming.

In case of PANI/AgPt, the impact of the bipolaron (1375 cm^−1^) band slightly increased, and the polaron band shifted from 1340 to 1350 cm^−1^. The peak related to N-H deformation in the semiquinonoid structures shifted from 1509 to 1513 cm^−1^, and the peak related to C–C stretching vibration in the semiquinonoid structures shifted from 1594 to 1603 cm^−1^. Moreover, the peak related to Ag-O vibrations shifted from 248 cm^−1^ to 262 cm^−1^, which indicated an increase in the interactions. All these changes in the spectrum testify to a slight shift in the electron density between NPs and PANI, thus pointing at the adsorption of NPs on PANI. No signs of PANI’s participation in the reduction process of metal ions were found.

Summarizing the results of Raman spectroscopy, we can conclude that for all the composites, the impact of the bipolaron band increased in comparison with pure PANI. This shown by charge localization increasing in the structure of the composites, which allowed the interaction between PANI and NPs. The data presented here also allowed us to draw the conclusion that in case of the deposition of platinum, PANI acted as a reducing agent, unlike composites with silver (PANI/Ag and PANI/AgPt).

**Figure 4:**
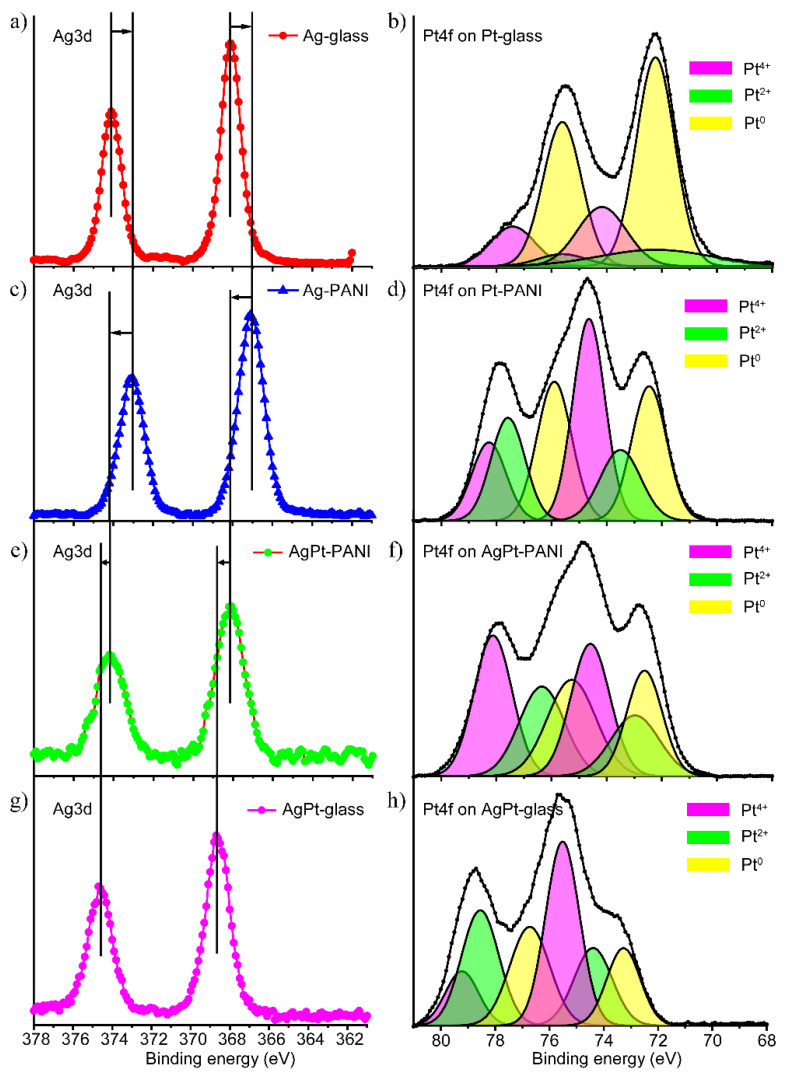
Left: XPS data for silver; right: XPS data for Pt. (**a**,**b**) Monometallic NPs on glass; (**c**,**d**) monometallic NPs on PANI; (**e**,**f**) bimetallic NPs on PANI; (**g**,**h**) bimetallic NPs on glass.

The effect of PANI on MNPs was also studied by XPS spectroscopy. Figure 4 shows the XPS spectra for Ag, Pt, and AgPt NPs. To evaluate the impact of PANI, additional data for NPs deposited on a glass substrate by LID are presented for comparison.

An analysis of the presented data showed that the presence of PANI influenced the signals of both silver and platinum. When comparing the spectra of monometallic silver NPs on glass and PANI (Figure 4a,c), one can see that the bands have shifted toward lower energies in the presence of PANI, which indicated the redistribution of the electron density between PANI and silver. At the same time, for the Ag3d signal in monometallic and bimetallic systems on PANI (Figure 4c,e) there was a reverse shift, indicating that the effect of PANI on silver in a bimetallic system was less than in the case of a monometallic one. A comparison of the position of the Ag3d signal in bimetallic NPs on PANI and on glass showed an insignificant shift in the presence of PANI (Figure 4e,g). Thus, the presence of PANI affects the position of silver lines in monometallic NPs but has almost no effect in case of bimetallic NPs.

In the Pt4f spectrum of the monometallic Pt sample (Figure 4b), in addition to the main metal peak of Pt^0^, there were components with higher binding energies, which may be associated with the oxidation states of platinum 2+ and 4+. The presence of PANI significantly affected the shape of the spectrum (Figure 4b,d) and resulted in an increase in the high-energy components. In addition, a shift in the peaks towards higher energies was observed, which indicates a shift in the electron density from PANI to platinum. When comparing the Pt4f spectra for monometallic and bimetallic NPs on PANI, one can see that despite no change in the position of the peaks in the total spectrum, there were shifts in the Pt^0^ and Pt^2+^ bands in deconvolution towards lower energies. The observed shifts indicated a redistribution of the electron density between Pt4f and Ag3d, and confirmed the formation of the Ag-Pt alloy phase. The formation of bimetallic AgPt NPs on glass was also observed previously and confirmed by TEM analysis [42].

The comparison of the Pt4f spectra for bimetallic NPs on PANI and glass (Figure 4f,h) showed that in presence of PANI, the position of the peaks shifted towards lower energies, and the contribution of low-energy components was higher. This shift indicates a redistribution of the electron density between PANI and platinum. The data demonstrate the effect of PANI on the formation of NPs. In the case of bimetallic NPs, the interaction between PANI and platinum was higher than that between PANI and silver.

Thus, the comprehensive analysis based on Raman spectroscopy, SEM, EDX, and XPS allowed us to suggest the following peculiarities of the nanocomposites’ formation, depending on the composition of the MNPs. For PANI/Ag, silver NPs formed as a result of LID, and the interaction of Ag NPs with PANI due to adsorption was observed. In the case of nanocomposite PANI/Pt, we can assume that PANI played a role in the reduction in Pt during LID processes. Formation of PANI/AgPt nanocomposites takes place via the following mechanism. As the first step, the formation of silver NPs took place through the photoinduced process of LID. Next, the galvanic exchange process occurs [57], in which silver NPs acted as reducing agent, resulting in partial replacement of Ag by Pt. The reduction potential of silver Ag^+^/Ag^0^ is +0.78 V, and the reduction potential of Pt^2+^/Pt^0^ is +1.2 V versus NHE, so this process is possible. If the galvanic exchange takes place, two silver atoms are replaced by one platinum atom. Therefore, the size of bimetallic NPs should be smaller than that of Ag NPs; this is exactly what we observed according to the SEM analysis in Figure 2a–c. In the process of galvanic exchange, silver acts as a reducing agent more effectively than PANI, which is why there is an insufficient interaction between PANI and Pt in a bimetallic system. This was reflected in the Raman and XPS measurements: the spectra of the PANI/Ag and PANI/AgPt composites were close to each other. Thus, the Raman spectroscopy and XPS data also testify in favor of the proposed mechanism.

### 3.2. Electrochemical Studies of the Composites

**Figure 5:**
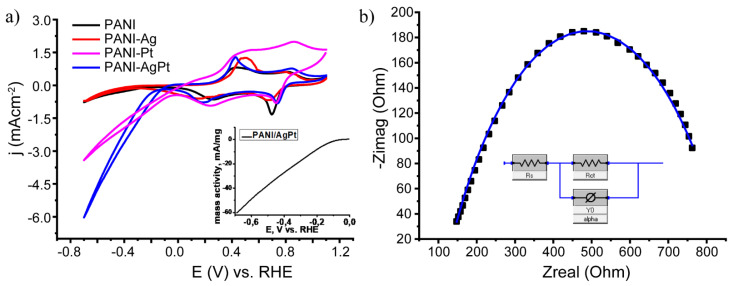
(**a**) CV of the obtained composites: PANI (black), PANI/Ag (red), PANI/Pt-1 (pink), and PANI/AgPt (blue); the insert shows the mass activity of the PANI/AgPt samples. (**b**) Impedance spectroscopy data for PANI/AgPt.

After structural characterization, the obtained PANI/Ag, PANI/Pt, and PANI/AgPt composites were studied by cyclic voltammetry. The results are presented in Figure 5a. The mass activity of the PANI/AgPt samples is presented in the insert in Figure 5a.

In the CV data presented here, pairs of oxidation and reduction waves typical of polyaniline could be observed: the leucoemeraldine–emeraldine transition (+0.1 V in the cathodic curve, +0.4 V in the anodic curve) and the emeraldine–pernigraniline transition (+0.7 V in the cathodic curve, +0.9V in the anodic curve). The hydrogen reduction signal (negative potential region) was insignificant for the PANI/Ag sample; however, it was pronounced for PANI/Pt. For this sample, the contribution of the capacitive current was observed, which may be related to the partial destruction/modification of PANI discussed above. This effect was leveled out for the bimetallic system, which also confirmed our previous conclusions. The start of the reaction on bimetallic samples was observed at an overpotential of ~150 mV. The current density in the case of a bimetallic system was higher than in the case of a PANI/Pt system; thus, a bimetallic system demonstrated higher activity than a monometallic one. This effect was caused by the presence of silver in the NPs’ composition [58], as no other parameters of the system were changed. The presence of silver changed the electronic structure of the NPs’ surface [59]. This led to a change in the adsorption energy of hydrogen [60] and may have been the reason for the higher activity of the bimetallic system. Moreover, the activity of various Pt sites may have changed in the presence of silver, which made the bimetallic system more effective. Additionally, as shown in Figure 2, the distribution of AgPt NPs on the surface of PANI was more uniform than that of Pt NPs. The combination of all the factors listed here may be the reason for the higher activity of bimetallic system in comparison with the monometallic one.

The obtained PANI/AgPt nanocomposite system was also studied by impedance spectroscopy (Figure 5b). The measurements were carried out at −0.5 V vs. an Ag/AgCl electrode, with amplitude fluctuations of ±10 mV/ms. The results and the fitting model are presented in Figure 5b. The uncompensated electrolyte resistance was 120.2 ohms. The value of resistance caused by charge transfer was R_ct_ = 736.6 ohms. The contribution of the diffusion component was negligible. We assumed that the reaction on the electrode is the limiting stage.

**Figure 6:**
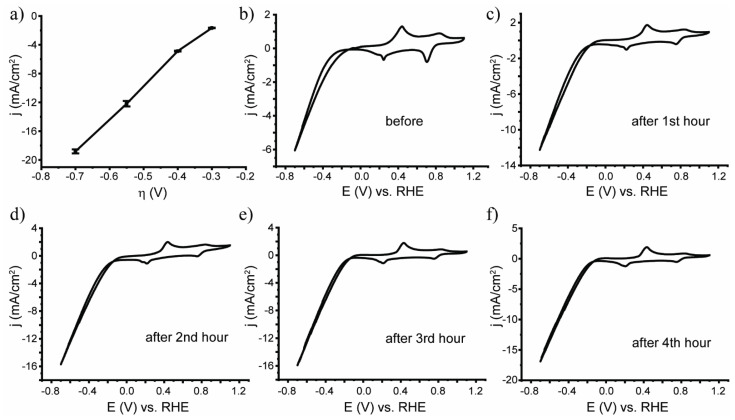
(**a**) Chronoamperometric data for PANI/AgPt at different overpotentials; (**b**–**f**) CV of PANI/AgPt samples recorded before and after each hour of chronoamperometry.

To study the stability of the bimetallic system, chronoamperometric measurements were carried out at various overpotentials. The results are presented in Figure 6a. As can be seen, the samples demonstrated high stability over a wide range of overpotentials. The relative change in the current during the measurements was not greater than 6%.

To identify the effect of the overvoltage applied during chronoamperometry on the structure of the samples, cyclic voltammograms were recorded after each step of the chronoamperometric measurements (Figure 6b–f). As can be seen from the data, the catalytic current increased significantly after the first (application of −0.3 V overpotential) and the second (application of −0.4 V overpotential) hours of chronoamperometric measurement, and almost did not change after the third (application of −0.55 V overpotential) and fourth (application of −0.7 V overpotential) hours. This indicated the electrochemical activation of the system at low overpotentials and demonstrated high stability at high overpotentials. After activation, a current density of 10 mA/cm^2^ was achieved at an overpotential of η = 0.50 V. It can be concluded that PANI/AgPt samples obtained by the LID method from a solution of the precursor C_7_H_5_AgO_2_+Pt(NH_3_)_4_(OH)_2_ are stable over a wide range of overvoltages. The destruction of neither metal NPs nor PANI was observed.

**Figure 7:**
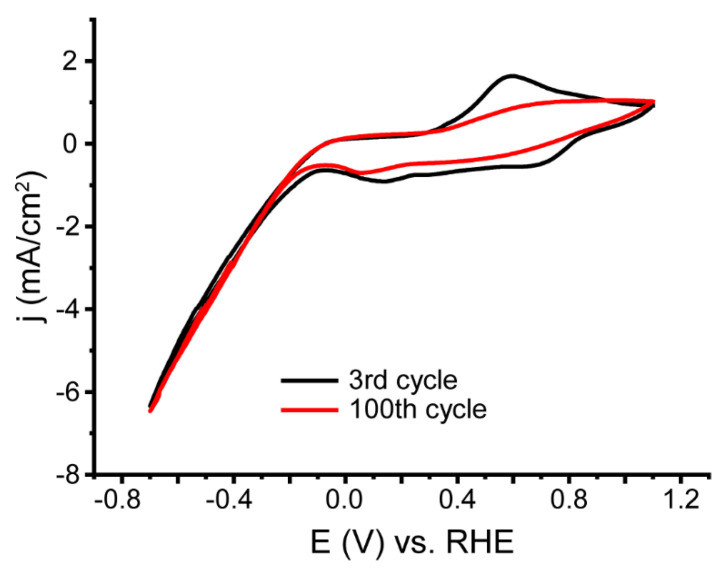
CV of the PANI/AgPt composite, 3rd and 100th cycles.

Additional stability measurements were carried out with continuous cycling. Figure 7 shows the CVs for Cycles 3 and 100 of the PANI/AgPt sample.

From the data presented here, it can be seen that the density of the catalytic current was stable, while changes were observed in the region of the redox transitions of PANI. In particular, the currents of the emeraldine–leucoemeraldine transition decreased. Thus, it can be concluded that the polymer matrix was modified during prolonged cycling, but the metal NPs were not washed out from its surface.

## 4. Conclusions

The current study presents a perspective approach to crating nanocomposite electrodes based on laser-induced deposition of metal NPs on polyaniline structures. Here, we performed LID of Ag, Pt, and bimetallic AgPt NPs from aqueous solutions of commercially available precursors on the surface of a PANI film synthesized in situ. Comprehensive characterization of the nanocomposites with different techniques (SEM, EDX, Raman spectroscopy, and XPS) allowed us to uncover the mechanisms of the formation of PANI/MNPs, which were found to be different, depending on the NPs’ composition. Thus, for PANI/Ag, silver NPs formed as a result of LID. The interaction of Ag NPs and PANI was demonstrated by the adsorption observed by the change in the Raman spectra of the PANI structure, which allowed us to infer its participation in the reduction in P. If we take the XRS data into account, this also indicates the interaction of PANI and Pt NPs. In the case of a bimetallic system, the process is more complex. As the first step, the formation of silver NPs occurred via a photoinduced process (LID). Next, a galvanic exchange process took place, where silver acted as a reducing agent and was partly replaced by Pt, resulting in formation of AgPt NPs.

We demonstrated that the LID of MNPs onto a polymer surface is not only possible but also allows PANI–MNP interactions. The interesting feature of LID on PANI is the participation of the substrate in the formation of the MNPs, which is of great importance for the creation of stable functional nanocomposites for electrochemical applications.

PANI/MNP composites were tested by electrocatalysis of the hydrogen evolution reaction. The PANI/AgPt composites demonstrated the onset overpotential of hydrogen evolution reaction at ~150 mV, leading to a current density of 10 mA/cm^2^ at an overpotential of η = 0.50 V, and showing the high stability of the metal component during continuous cycling.

As LID can be realized using an extensive list of metals, the presented approach to the formation of stable electrocatalytically active nanocomposite electrodes is universal, as it can be extended to various electrochemical reactions and applications.

## Figures and Tables

**Table 1:** Main characteristic vibration bands of the synthesized PANI.

Band, cm^−1^	Vibration	Reference
420	Out-of-plane ring deformation	[49,50]
520	Out-of-plane ring deformation	[49]
572	Vibration of phenoxazine-type units	[49,51,52]
720	Amine deformations reported for the bipolaronic form of emeraldine salt	[49]
780 shoulder	Ring deformation in the emeraldine base	[53]
808	Substituted benzene ring deformations;out-of-plane C-H deformation of thequinonoid ring; ring deformation of the SQ ring	[48,49]
865 shoulder	Substituted benzene ring deformations	[49]
1170	Bending vibrations involving C-H bonds in semiquinonoid rings	[48,49,50]
1254	C–N stretching in quinonoid structures	[47,53]
1335	Polaron-associated vibrational mode C-N^+.^	[50,53,54]
1373	Bipolaron	[53,54]
1506	N-H deformation in the semiquinonoid structures	[50,53]
1595	C–C stretching vibration in the semiquinonoid structures	[48,50,53]

## Data Availability

Not applicable.

## References

[1] Zhao G., Rui K., Dou S.X., Sun W. (2018). Heterostructures for Electrochemical Hydrogen Evolution Reaction: A Review. Adv. Funct. Mater..

[2] Yu F., Zhou H., Huang Y., Sun J., Qin F., Bao J., Goddard W.A., Chen S., Ren Z. (2018). High-Performance Bifunctional Porous Non-Noble Metal Phosphide Catalyst for Overall Water Splitting. Nat. Commun..

[3] Li J., Banis M.N., Ren Z., Adair K.R., Doyle-Davis K., Meira D.M., Finfrock Y.Z., Zhang L., Kong F., Sham T. (2021). Unveiling the Nature of Pt Single-Atom Catalyst during Electrocatalytic Hydrogen Evolution and Oxygen Reduction Reactions. Small.

[4] Nazir R., Fageria P., Basu M., Gangopadhyay S., Pande S. (2017). Decoration of Pd and Pt Nanoparticles on a Carbon Nitride (C 3 N 4) Surface for Nitro-Compounds Reduction and Hydrogen Evolution Reaction. New J. Chem..

[5] Abbaspour A., Mirahmadi E. (2013). Electrocatalytic Hydrogen Evolution Reaction on Carbon Paste Electrode Modified with Ni Ferrite Nanoparticles. Fuel.

[6] Wan W., Wei S., Li J., Triana C.A., Zhou Y., Patzke G.R. (2019). Transition Metal Electrocatalysts Encapsulated into N-Doped Carbon Nanotubes on Reduced Graphene Oxide Nanosheets: Efficient Water Splitting through Synergistic Effects. J. Mater. Chem. A.

[7] Lang L., Shi Y., Wang J., Wang F.-B., Xia X.-H. (2015). Hollow Core–shell Structured Ni–Sn@ C Nanoparticles: A Novel Electrocatalyst for the Hydrogen Evolution Reaction. ACS Appl. Mater. Interfaces.

[8] Wang J., Zhu H., Chen J., Zhang B., Zhang M., Wang L., Du M. (2016). Small and Well-Dispersed Cu Nanoparticles on Carbon Nanofibers: Self-Supported Electrode Materials for Efficient Hydrogen Evolution Reaction. Int. J. Hydrogen Energy.

[9] Cao S., Jiang J., Zhu B., Yu J. (2016). Shape-Dependent Photocatalytic Hydrogen Evolution Activity over a Pt Nanoparticle Coupled gC 3 N 4 Photocatalyst. Phys. Chem. Chem. Phys..

[10] Li Z., Qi Z., Wang S., Ma T., Zhou L., Wu Z., Luan X., Lin F.-Y., Chen M., Miller J.T. (2019). In Situ Formed Pt3Ti Nanoparticles on a Two-Dimensional Transition Metal Carbide (MXene) Used as Efficient Catalysts for Hydrogen Evolution Reactions. Nano Lett..

[11] Jiang L.-Y., Huang X.-Y., Wang A.-J., Li X.-S., Yuan J., Feng J.-J. (2017). Facile Solvothermal Synthesis of Pt 76 Co 24 Nanomyriapods for Efficient Electrocatalysis. J. Mater. Chem. A.

[12] Li J., Sun S. (2019). Intermetallic Nanoparticles: Synthetic Control and Their Enhanced Electrocatalysis. Acc. Chem. Res..

[13] Xiao M., Liang X., Li W., Yang Y., Miao Y. (2015). Synthesis of Ultrafine Pt/Pd Bimetallic Nanoparticles and Their Decoration on MWCNTs for Hydrogen Evolution. J. Electrochem. Soc..

[14] Mourdikoudis S., Regner J., Gusmão R., Sofer Z. (2022). Bismuth-Noble Metal Alloy Nanostructures Prepared by Colloidal Chemical Routes for Use in Hydrogen Evolution and Oxygen Reduction Electrocatalysis: Bi-Pt Versus Bi-Pd. Adv. Sustain. Syst..

[15] Huang H., Fu L., Kong W., Ma H., Zhang X., Cai J., Wang S., Xie Z., Xie S. (2022). Equilibrated PtIr/IrOx Atomic Heterojunctions on Ultrafine 1D Nanowires Enable Superior Dual-Electrocatalysis for Overall Water Splitting. Small.

[16] Pelicano C.M., Saruyama M., Takahata R., Sato R., Kitahama Y., Matsuzaki H., Yamada T., Hisatomi T., Domen K., Teranishi T. (2022). Bimetallic Synergy in Ultrafine Cocatalyst Alloy Nanoparticles for Efficient Photocatalytic Water Splitting. Adv. Funct. Mater..

[17] Zou Y., Goei R., Ong S.A., Ong A.J., Huang J., Tok A.I.Y. (2022). Development of Core-Shell Rh@ Pt and Rh@ Ir Nanoparticle Thin Film Using Atomic Layer Deposition for HER Electrocatalysis Applications. Processes.

[18] Zhang E., Ma F., Liu J., Sun J., Chen W., Rong H., Zhu X., Liu J., Xu M., Zhuang Z. (2018). Porous Platinum–silver Bimetallic Alloys: Surface Composition and Strain Tunability toward Enhanced Electrocatalysis. Nanoscale.

[19] Belgherbi O., Chouder D., Lakhdari D., Dehchar C., Laidoudi S., Lamiri L., Hamam A., Seid L. (2020). Enzyme-Free Glucose Sensor Based on Star-Like Copper Particles-Polyaniline Composite Film. J. Inorg. Organomet. Polym. Mater..

[20] Salahandish R., Ghaffarinejad A., Naghib S.M., Niyazi A., Majidzadeh-A K., Janmaleki M., Sanati-Nezhad A. (2019). Sandwich-Structured Nanoparticles-Grafted Functionalized Graphene Based 3D Nanocomposites for High-Performance Biosensors to Detect Ascorbic Acid Biomolecule. Sci. Rep..

[21] Chu T.-X., Vu V.-P., Tran H.-T., Tran T.-L., Tran Q.-T., Manh T. (2020). Le Molecularly Imprinted Polyaniline Nanowire-Based Electrochemical Biosensor for Chloramphenicol Detection: A Kinetic Study of Aniline Electropolymerization. J. Electrochem. Soc..

[22] Oueiny C., Berlioz S., Perrin F. (2013). Carbon Nanotube–polyaniline Composites Review Article. Prog. Polym. Sci..

[23] Tabrizi A.G., Arsalani N., Mohammadi A., Ghadimi L.S., Ahadzadeh I., Namazi H. (2018). A New Route for the Synthesis of Polyaniline Nanoarrays on Graphene Oxide for High-Performance Supercapacitors. Electrochim. Acta.

[24] Zhang M., Wang X., Yang T., Zhang P., Wei X., Zhang L., Li H. (2020). Polyaniline/graphene Hybrid Fibers as Electrodes for Flexible Supercapacitors. Synth. Met..

[25] Ganesan R., Gedanken A. (2008). Organic–inorganic Hybrid Materials Based on polyaniline/TiO2 Nanocomposites for Ascorbic Acid Fuel Cell Systems. Nanotechnology.

[26] Mondal S.K., Raman R.K., Shukla A.K., Munichandraiah N. (2005). Electrooxidation of Ascorbic Acid on Polyaniline and Its Implications to Fuel Cells. J. Power Sources.

[27] Ding L., Su B. (2015). A Non-Enzymatic Hydrogen Peroxide Sensor Based on Platinum Nanoparticle–polyaniline Nanocomposites Hosted in Mesoporous Silica Film. J. Electroanal. Chem..

[28] Lakhdari D., Guittoum A., Benbrahim N., Belgherbi O., Berkani M., Vasseghian Y., Lakhdari N. (2021). A Novel Non-Enzymatic Glucose Sensor Based on NiFe (NPs)–polyaniline Hybrid Materials. Food Chem. Toxicol..

[29] Chiu W.-T., Chang T.-F.M., Sone M., Tixier-Mita A., Toshiyoshi H. (2020). Roles of TiO2 in the Highly Robust Au Nanoparticles-TiO2 Modified Polyaniline Electrode towards Non-Enzymatic Sensing of Glucose. Talanta.

[30] Essousi H., Barhoumi H., Bibani M., Ktari N., Wendler F., Al-Hamry A., Kanoun O. (2019). Ion-Imprinted Electrochemical Sensor Based on Copper Nanoparticles-Polyaniline Matrix for Nitrate Detection. J. Sensors.

[31] Manshina A.A., Grachova E.V., Povolotskiy A.V., Povolotckaia A.V., Petrov Y.V., Koshevoy I.O., Makarova A.A., Vyalikh D.V., Tunik S.P. (2015). Laser-Induced Transformation of Supramolecular Complexes: Approach to Controlled Formation of Hybrid Multi-Yolk-Shell Au-Ag@a-C:H Nanostructures. Sci. Rep..

[32] Avilova E.A., Khairullina E.M., Shishov A.Y., Eltysheva E.A., Mikhailovskii V., Sinev D.A., Tumkin I.I. (2022). Direct Laser Writing of Copper Micropatterns from Deep Eutectic Solvents Using Pulsed near-IR Radiation. Nanomaterials.

[33] Khairullina E., Ratautas K., Panov M., Andriianov V., Mickus S., Manshina A., Račiukaitis G., Tumkin I. (2022). Laser-Assisted Surface Activation for the Fabrication of Flexible Non-Enzymatic Cu-Based Sensors. Mikrochim Acta.

[34] Schlicht S., Kireev A., Vasileva A., Grachova E.V., Tunik S.P., Manshina A.A., Bachmann J. (2017). A Model Electrode of Well-Defined Geometry Prepared by Direct Laser-Induced Decoration of Nanoporous Templates with Au-Ag@C Nanoparticles. Nanotechnology.

[35] Vasileva A., Haschke S., Mikhailovskii V., Gitlina A., Bachmann J., Manshina A. (2020). Direct Laser-Induced Deposition of AgPt@ C Nanoparticles on 2D and 3D Substrates for Electrocatalytic Glucose Oxidation. Nano-Struct. Nano-Objects.

[36] Manshina A., Ivanova T., Povolotskiy A. (2010). Laser-Induced Deposition of Hetero-Metallic Structures from Liquid Phase. Laser Phys..

[37] Manshina A.a., Povolotskiy A.V., Povolotskaya A.V., Ivanova T.Y., Koshevoy I.O., Tunik S.P., Suvanto M., Pakkanen T.a. (2012). Laser-Induced Heterometallic Phase Deposition from Solutions of Supramolecular Complexes. Surf. Coatings Technol..

[38] Manshina A., Povolotskiy A., Ivanova T., Kurochkin A.Y., Tver’yanovich Y., Kim D., Kim M., Kwon S. (2007). Laser-assisted metal deposition from CuSO4-based electrolyte solution. Laser Phys. Lett.

[39] Man’shina A.A., Povolotskiy A.V., Ivanova T.Y., Kurochkin A.V., Tver’yanovich Y.S., Kim D., Kim M., Kwon S. (2007). Laser-Induced Copper Deposition on the Surface of an Oxide Glass from an Electrolyte Solution. Glas. Phys. Chem..

[40] Bashouti M.Y., Povolotckaia A.V., Povolotskiy A.V., Tunik S.P., Christiansen S.H., Leuchs G., Manshina A.A. (2016). Spatially-Controlled Laser-Induced Decoration of 2D and 3D Substrates with Plasmonic Nanoparticles. RSC Adv..

[41] Haschke S., Pankin D., Mikhailovskii V., Barr M.K.S., Both-Engel A., Manshina A., Bachmann J. (2019). Nanoporous Water Oxidation Electrodes with a Low Loading of Laser-Deposited Ru/C Exhibit Enhanced Corrosion Stability. Beilstein J. Nanotechnol..

[42] Mamonova D.V., Vasileva A.A., Petrov Y.V., Koroleva A.V., Danilov D.V., Kolesnikov I.E., Bikbaeva G.I., Bachmann J., Manshina A.A. (2021). Single Step Laser-Induced Deposition of Plasmonic Au, Ag, Pt Mono-, Bi-and Tri-Metallic Nanoparticles. Nanomaterials.

[43] Armarego W.L.F. (2017). Purification of Laboratory Chemicals.

[44] Mamonova D.V., Vasileva A.A., Petrov Y.V., Danilov D.V., Kolesnikov I.E., Kalinichev A.A., Bachmann J., Manshina A.A. (2020). Laser-Induced Deposition of Plasmonic Ag and Pt Nanoparticles, and Periodic Arrays. Materials..

[45] Vasileva A., Pankin D., Mikhailovskii V., Kolesnikov I., Mínguez-Bacho I., Bachmann J., Manshina A. (2021). In Situ Microsynthesis of Polyaniline: Synthesis–structure–conductivity Correlation. New J. Chem..

[46] Cochet M., Louarn G., Quillard S., Buisson J.P., Lefrant S. (2000). Theoretical and Experimental Vibrational Study of Emeraldine in Salt Form. Part II. J. Raman Spectrosc..

[47] Lindfors T., Ivaska A. (2005). Raman Based pH Measurements with Polyaniline. J. Electroanal. Chem..

[48] Ciric-Marjanovic G., Dragicevic L., Milojevic M., Mojovic M., Mentus S., Dojcinovic B., Marjanovic B., Stejskal J. (2009). Synthesis and Characterization of Self-Assembled Polyaniline Nanotubes/silica Nanocomposites. J. Phys. Chem. B.

[49] Trchová M., Morávková Z., Bláha M., Stejskal J. (2014). Raman Spectroscopy of Polyaniline and Oligoaniline Thin Films. Electrochim. Acta.

[50] 50. Rohom A.B., Londhe P.U., Mahapatra S.K., Kulkarni S.K., Chaure N.B. (2014). Electropolymerization of polyaniline thin films. High Perform. Polym..

[51] Nobrega M.M., Martins V.L., Torresi R.M., Temperini M.L.A. (2014). One-Step Synthesis, Characterization, and Properties of Emeraldine Salt Nanofibers Containing Gold Nanoparticles. J. Phys. Chem. C.

[52] Rozlivkova Z., Trchova M., Exnerova M., Stejskal J. (2011). The Carbonization of Granular Polyaniline to Produce Nitrogen-Containing Carbon. Synth. Met..

[53] Morávková Z., Bober P. (2018). Writing in a Polyaniline Film with Laser Beam and Stability of the Record: A Raman Spectroscopy Study. Int. J. Polym. Sci..

[54] Chakraborty P., Kothari A., Nagarajan R. (2018). Highly Ordered Polyaniline as an Efficient Dye Remover. Adsorpt. Sci. Technol..

[55] Song E., Choi J.-W. (2013). Conducting Polyaniline Nanowire and Its Applications in Chemiresistive Sensing. Nanomaterials.

[56] López I.A., Ceballos M., Hernández G., Acosta LGómez I. (2015). Silver Nanoprisms and Nanospheres for Prosthetic Biomaterials. Revista Mexicana de Física.

[57] Nazir R., Fageria P., Basu M., Pande S. (2017). Decoration of Carbon Nitride Surface with Bimetallic Nanoparticles (Ag/Pt, Ag/Pd, and Ag/Au) via Galvanic Exchange for Hydrogen Evolution Reaction. J. Phys. Chem. C.

[58] Liu Q., He Y.M., Weng X., Wang A.J., Yuan P.X., Fang K.M., Feng J.J. (2018). One-pot aqueous fabrication of reduced graphene oxide supported porous PtAg alloy nanoflowers to greatly boost catalytic performances for oxygen reduction and hydrogen evolution. J. Colloid Interface Sci..

[59] Lv J.J., Feng J.X., Li S.S., Wang Y.Y., Wang A.J., Zhang Q.L., Feng J.J. (2014). Ionic liquid crystal-assisted synthesis of PtAg nanoflowers on reduced graphene oxide and their enhanced electrocatalytic activity toward oxygen reduction reaction. Electrochim. Acta.

[60] Weber I., Solla-Gullon J., Brimaud S., Feliu J.M., Behm R.J. (2017). Structure, surface chemistry and electrochemical de-alloying of bimetallic PtxAg100-x nanoparticles: Quantifying the changes in the surface properties for adsorption and electrocatalytic transformation upon selective Ag removal. J. Electroanal. Chem..

